# Novel 3′ Proximal Replication Elements in Umbravirus Genomes

**DOI:** 10.3390/v14122615

**Published:** 2022-11-23

**Authors:** Philip Z. Johnson, Hannah M. Reuning, Sayanta Bera, Feng Gao, Zhiyou Du, Anne E. Simon

**Affiliations:** Department of Cell Biology and Molecular Genetics, University of Maryland, College Park, MD 20742, USA

**Keywords:** umbravirus, pea enation mosaic virus 2, positive-strand RNA virus replication and translation, RNA structure/function

## Abstract

The 3′ untranslated regions (UTRs) of positive-strand RNA plant viruses commonly contain elements that promote viral replication and translation. The ~700 nt 3′UTR of umbravirus pea enation mosaic virus 2 (PEMV2) contains three 3′ cap-independent translation enhancers (3′CITEs), including one (PTE) found in members of several genera in the family *Tombusviridae* and another (the 3′TSS) found in numerous umbraviruses and several carmoviruses. In addition, three 3′ terminal replication elements are found in nearly every umbravirus and carmovirus. For this report, we have identified a set of three hairpins and a putative pseudoknot, collectively termed “Trio”, that are exclusively found in a subset of umbraviruses and are located just upstream of the 3′TSS. Modification of these elements had no impact on viral translation in wheat germ extracts or in translation of luciferase reporter constructs in vivo. In contrast, Trio hairpins were critical for viral RNA accumulation in *Arabidopsis thaliana* protoplasts and for replication of a non-autonomously replicating replicon using a trans-replication system in *Nicotiana benthamiana* leaves. Trio and other 3′ terminal elements involved in viral replication are highly conserved in umbraviruses possessing different classes of upstream 3′CITEs, suggesting conservation of replication mechanisms among umbraviruses despite variation in mechanisms for translation enhancement.

## 1. Introduction

The 3′ untranslated regions (UTRs) of positive-strand RNA [(+)RNA] viruses commonly contain elements that promote minus (−)-strand synthesis and translation [1,2,3]. These elements exhibit a variety of secondary and tertiary structural motifs such as stem-loops and pseudoknots and some engage in long-distance intragenomic RNA-RNA interactions to support translation initiating at the 5′end as well as internal ribosomal recoding events [1,4]. Elements supporting translation are known to bind translation initiation factors or ribosomes/ribosomal subunits [4,5] whereas elements important for replication bind proteins such as the viral RNA-dependent RNA polymerase (RdRp) and other components of the viral replicase complex including various host proteins involved in viral replication [1,6,7,8].

**Figure 1:**
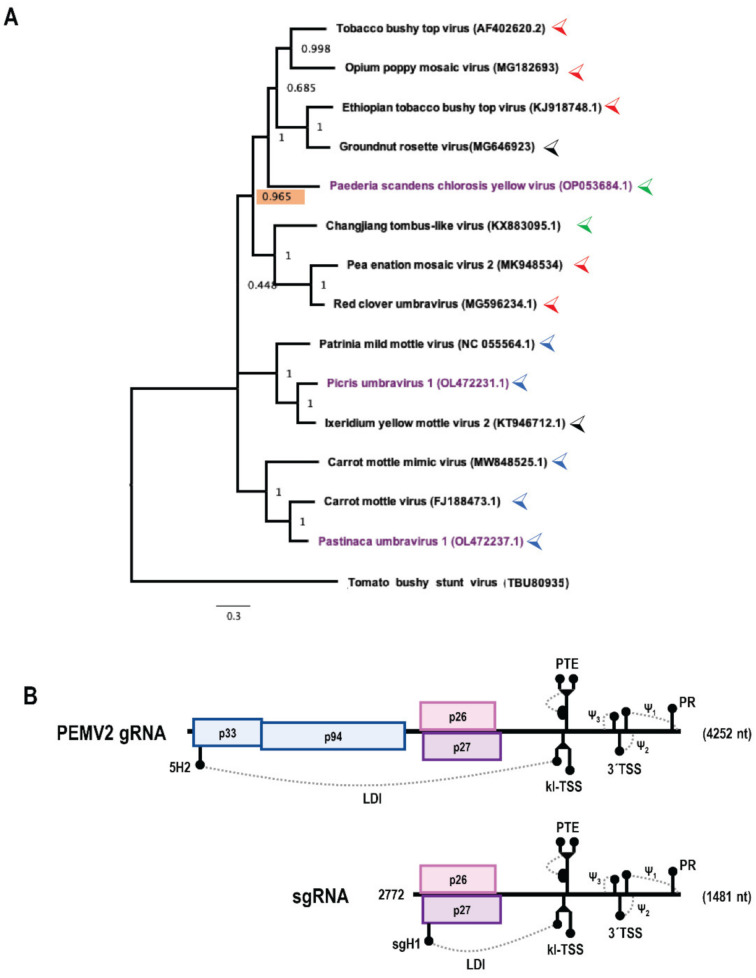
Umbravirus phylogenetic tree and pea enation mosaic virus 2 (PEMV2) genome organization. (**A**) Maximum likelihood phylogenetic tree of umbraviruses based on RNA-dependent RNA polymerase (RdRp) nucleotide sequences. Tomato bushy stunt virus (TBSV) RdRp sequence (TBU80935) was used as an outgroup. Values next to each node indicate confidence probability in the node (out of 1000 bootstraps in building the tree). Scale bar indicates nucleotide substitutions per site. Purple color denotes three recently discovered umbraviruses (paederia scandens chlorosis yellow virus [PSCYV], picris umbravirus 1 [PicUV1] and pastinaca umbravirus 1 [PasUV1]). Colored carets next to viruses indicate the presence of complete Trio elements upstream of the 3′ T-shaped structure (TSS) (red), incomplete Trio elements upstream of the 3′TSS (blue), no Trio elements upstream of the 3′TSS (green) or entirely divergent 3′ terminal regions (black). Confidence probability for the nearest ancestral node for the 5 umbraviruses possessing complete Trio elements is highlighted in salmon color. (**B**) PEMV2 genomic (g)RNA and subgenomic (sg)RNA. The gRNA is 4252 nt long and the sgRNA is composed of the 3′ 1481 nt of the gRNA sequence (beginning at position 2772). p33 and p94 are expressed from the gRNA and are required for replication and form the viral replicase complex. p26 and p27 are expressed from the sgRNA and are involved in movement of the virus within a host plant. Known 3′ untranslated region (UTR) elements are shown.

Cis-acting replication elements in the 3′UTRs of members of the family *Tombusviridae* have been intensively studied using turnip crinkle virus (TCV) and its satellite satC as models for the genus Carmovirus [3], and tomato bushy stunt virus (TBSV) as a model for the genus Tombusvirus [9]. Despite the close evolutionary relationship of replicase components between carmoviruses and viruses in the genus Umbravirus, which are also members of the family *Tombusviridae*, no information is currently available on replication elements in the 3′UTRs of umbraviruses. The genus Umbravirus is currently composed of 14 viruses, including three that were recently added to GenBank (Figure 1A), and all require the association with a helper virus to complete their infection cycle as they do not encode their own capsid protein or silencing suppressor [10]. Pea enation mosaic virus 2 (PEMV2) is a typical umbravirus with a 4252 nt genome encoding four proteins (Figure 1B). p94, the RdRp for PEMV2, is an extension product of p33 that is produced by a −1 programmed ribosomal frameshift event at the p33 stop codon [11]. p26 and p27, expressed from a subgenomic (sg)RNA, have overlapping open reading frames (ORFs). p26 is involved in long-distance movement of the virus through the vascular system of a host plant and additionally functions as a stabilizing protein [10], conferring protection against nonsense-mediated decay [12]. p27 facilitates cell-to-cell movement of the virus, as shown by studies of the corresponding protein in umbraviruses groundnut rosette virus (GRV) and carrot mottle virus (CMoV) [13,14]. PEMV2 is capable of replicating independently in single cells [15] and can systemically infect host plants including *Nicotiana benthamiana* [16], but requires the helper enamovirus PEMV1 for plant-to-plant transmission.

A characteristic feature of umbravirus genomes is an exceptionally long 3′UTR (~700 nt) compared with other tombusvirids. The 3′UTR of PEMV2 is densely packed with RNA structures, several of which have been shown to be required for efficient translation of its uncapped and non-polyadenylated genome (Figure 1B) [17,18]. For example, the kissing-loop T-shaped structure (kl-TSS) binds to ribosomal subunits and engages in a long-distance interaction with the terminal loop of a hairpin near the 5′end of the viral genome [19]. Just downstream of the kl-TSS is a panicum mosaic virus-like translation enhancer (PTE) that binds to the cap-binding translation initiation factor eIF4E [20,21] and thus sequesters co-complexed eIF4G. The kl-TSS and the PTE are the key translation enhancers for the genomic (g)RNA of PEMV2 [18], whereas the sgRNA also uses a 3′ proximal translation enhancer, known as the 3′TSS, which binds to 60S subunits and 80S ribosomes [17,22]. The 3′TSS, which is also found in a similar 3′ proximal location in the majority of umbraviruses as well as in carmoviruses TCV and cardamine chlorotic fleck virus, is composed of hairpins H4a, H4b and H5 and pseudoknots Ψ_2_ and Ψ_3_ [23]. These hairpins and pseudoknots are also present in untranslated satC, and all are important for replication of satC with the exception of Ψ_3_ [23,24]. PEMV2 and TCV/satC also contain a 3′ terminal hairpin known as “PR” that serves as the satC core promoter for (−)-strand synthesis [25,26,27]. H5 and PR are present in nearly all carmoviruses and umbraviruses with the only exceptions being umbraviruses whose 3′ terminal sequences may be missing or incorrectly sequenced, such as GRV, ixeridium yellow mottle virus (IXYMoV) and picris umbravirus 1 (PicUV1). The PEMV2 and TCV PR terminal loops engage in a long-distance interaction with the bulge loop of a hairpin located at the recoding site just downstream of the 5′ proximal ORF stop codon [11,28,29]. Downstream of the PR in nearly all umbraviruses and carmoviruses (including PEMV2, TCV and satC) is the sequence 5′GCCC-OH, which forms a pseudoknot (Ψ_1_) with hairpin H5 apical loop (umbraviruses) or symmetrical internal loop (carmoviruses) [30,31]. The structural similarity between the 3′ terminal regions of PEMV2 and TCV suggests similar mechanisms for replication of carmoviruses and umbraviruses.

Despite our growing understanding of how the 3′UTR of PEMV2 contributes to translation, no replication elements upstream of the 3′TSS have been identified for any umbravirus. Here, we report the identification of three hairpins and a putative pseudoknot specific to umbraviruses just upstream of the 3′TSS that play no discernible role in translation but are critical for viral accumulation in single cells in vivo, and for amplification of a PEMV2 replicon in planta that depends on exogenously supplied viral replication components. Similar conserved hairpins and pseudoknots are found in some, but not all, umbraviruses, and are absent from the 3′UTR of carmoviruses. These components thus represent the first known umbravirus-specific cis-acting replication elements.

## 2. Materials and Methods

### 2.1. Construction of Umbravirus Phylogenetic Trees

The RdRp and 3′UTR nucleotide sequences of umbraviruses were queried using Geneious software version 2022.2.2 at http://www.geneious.com (accessed on 10 October 2022) and GenBank IDs shown in Figure 1A and Supplemental Figure S3. Maximum likelihood phylogenetic trees were built using MEGA 11 software [32] using the TBSV RdRp sequence (TBU80935) as an outgroup for the tree in Figure 1B. After the multiple sequence alignment step of tree construction, sequences were trimmed to equal length. The trimmed multiple sequence alignment was then used to predict the best model to generate the tree (general time-reversible substitution model with a Gamma distribution; 1000 bootstraps). Phylogenetic trees were formatted using FigTree software version 1.4.4 at http://tree.bio.ed.ac.uk/software/figtree/ (accessed on 10 October 2022).

### 2.2. Oligonucleotide-Mediated Site-Directed Mutagenesis of PEMV2 Constructs and In Vitro Transcription of PEMV2 RNA

Oligonucleotide-mediated site-directed mutagenesis [33] was used to introduce mutations into expression constructs containing cDNA of PEMV2 full-length gRNA (pUC19-PEMV2) or sgRNA (pUC19-PEMV2sg). During the initial PCR step, Q5 high-fidelity DNA polymerase [New England BioLabs (NEB)] was used, and the number of PCR amplification cycles was kept to 12 cycles or less. In vitro transcription of PEMV2 gRNA using T7 RNA polymerase was performed as previously described [34] using pUC19-PEMV2 or pUC19-PEMV2sg plasmids linearized with PvuII (for high-throughput SHAPE of PEMV2 gRNA) or SmaI (for all other experiments involving in vitro transcribed PEMV2 transcripts). As described previously [17], PEMV2 gRNA transcripts produced from PvuII-linearized pUC19-PEMV2 contain 214 additional nt of vector sequence at the 3′end that provide the binding site for the probe used to determine the SHAPE reactivity of the 3′ terminus of PEMV2 gRNA.

### 2.3. High-Throughput SHAPE (Selective 2′ Hydroxyl Acylation Analyzed by Primer Extension) Structure Probing

All PEMV2 gRNA transcripts used in high-throughput SHAPE probing were transcribed from pUC19-PEMV2 template linearized with PvuII. As described previously [35], gRNA transcripts (12 pmol) were denatured at 65 °C for 3 min and then immediately cooled on ice for 2 min. The denatured RNA was then folded in buffer containing 80 mM Tris–HCl (pH 8.0), 11 mM Mg(CH_3_COO)_2_, and 160 mM NH_4_Cl at 37 °C for 10 min. After folding, reactions were divided in half: 3.25 µL of 100 mM of NMIA dissolved in dimethyl sulfoxide (DMSO) was added to the (+) reaction, while 3.25 µL of DMSO alone was added to the (−) negative modification control. (+) and (−) reactions were incubated for 30 min at 37 °C, ethanol precipitated, and then resuspended in 11 µL of ddH_2_O prior to reverse transcription. Primers used for reverse transcription were: PEMV2_R1: 5′-AGGAAACAGCTATGACC (positions 4319 to 4302) and PEMV2_R2: 5′-CGCGTTTGTGATCTTTTTGG (positions 3959 to 3940). Note that the final position of PEMV2 gRNA sequence is 4252, meaning that PEMV2_R1 binds within the 214 nt vector sequence present in transcripts due to linearization of pUC19-PEMV2 plasmid with PvuII prior to in vitro transcription. For reverse transcription reactions, 2.5 µM of PET-labeled or 6-carboxyfluorescein (FAM)-labeled oligonucleotides (PEMV_R1, PEMV_R2) were mixed with 6 pmoles of unmodified RNA (for dideoxynucleotide-based sequencing reactions) or NMIA- and DMSO-treated RNA [(+) and (−) reactions]. SuperScript III reverse transcriptase (Invitrogen) was used for reverse transcription reactions as described previously [36]. cDNA products were submitted to Genewiz (Chelmsford, MA, USA) for fragment analysis and resulting chromatograms were analyzed and peak measurements obtained using QuShape software version 1.0 [37].

### 2.4. Transfection of Protoplasts and RNA Gel Blots for Viral RNA Accumulation

Approximately 5 × 10^6^ protoplasts prepared from callus cultures of *A. thaliana* (ecotype Col-0) were transfected using polyethylene glycol with 20 to 25 μg of in vitro transcribed PEMV2 gRNA as described previously [38,39]. Mock samples were transfected with transfection solution alone. Infected protoplasts were incubated for 24 h at 22 °C in the dark. Total RNA was extracted using phenol-chloroform and RNA extraction buffer (50 mM Tris-HCl [pH 7.5], 5 mM EDTA [pH 8.0], 100 mM NaCl, 1% SDS), followed by ethanol precipitation. For RNA gel blots, 2 to 3 μg of total RNA was subjected to electrophoresis through an ethidium-stained, 1% nondenaturing agarose gel. 28S ribosomal RNA (rRNA) was used as a loading control. RNA was transferred from the gel to a positively charged nylon membrane by capillary transfer. Three different [γ-32P]dATP-labeled DNA probes (positions 2731 to 2771; 2969 to 3004; 3229 to 3270) complementary to the subgenomic portion of (+)-strand PEMV2 genome were used for hybridization at 50 °C. Blots were imaged using an Amersham Typhoon Biomolecular Imager. Intensities of gRNA bands on blots were quantified using ImageJ software version 1.53 [40].

### 2.5. In Vitro Translation of PEMV2 gRNA/sgRNA Transcripts in WGE

Wheat germ extract (5 μL; Promega) was programmed with 0.5 pmol of in vitro transcribed PEMV2 gRNA or sgRNA and [35S] methionine in a 10 μL reaction and incubated at 25 °C for 30 min. Translation reaction mixtures were resolved by 10% SDS-PAGE. Gels were dried, exposed to a phosphor screen, and visualized by autoradiography. Quantitation of band intensities was performed using ImageJ software.

### 2.6. In Vivo Luciferase Translation Assays

To assay for translation of PEMV2 gRNA in protoplasts, a luciferase reporter construct was used containing the 3′UTR (positions 3550 to 4252) and 5′ 89 nt of the gRNA [41]. For translation of p26, the 5′ 156 nt of the sgRNA (positions 2772 to 2927) replaced the 5′ 89 nt gRNA sequence, and for p27 translation, the 5′ 157 nt of the sgRNA was used [22]. Mutations were introduced into luciferase reporter constructs by oligonucleotide-mediated site-directed mutagenesis. Protoplasts of *A. thaliana* callus cultures were transfected with 30 μg of in vitro transcribed luciferase reporter construct RNA. Samples were also co-transfected with 10 μg of in vitro transcribed Renilla luciferase reporter construct RNA as a transfection control. Transfected protoplasts were incubated for 18 h at 22 °C under light. Luciferase levels were measured using a Modulus microplate multimode reader (Turner BioSystems, Sunnyvale, CA, USA) as described previously [42].

### 2.7. Trans-Replication Assay

PEMV2 gRNA cDNA containing mutations in the p33 start codon (AUG to ACG) and the slippery site of the −1 programed ribosomal frameshift signal just upstream of the p33 stop codon (GGAUUUU to GGAUUCU) [11], was inserted into Agrobacteria binary vector pCB301, generating pCB301-PEMV2(-p33/-p94). Ligation-independent cloning [43] was used to insert p33 and p94 ORFs into pCB301, generating constructs pCB301-p33 and pCB301-p94, which each included an upstream cauliflower mosaic virus 35S promoter. Since RNA transcribed from the viral replicon does not translate p33 and p94, viral replication is dependent on p33 and p94 expressed in trans from co-infiltrated pCB301-p33 and pCB301-p94. An additional construct encoding PoLV p14 in pBIN61S (pBIN61S-p14) was also concurrently infiltrated [44]. All T-DNA clones were separately transformed into *A. tumefaciens* GV3101 by electroporation [45], except for pBIN61S-p14, which was already present in *A. tumefaciens* C58C1. *A. tumefaciens* cultures were grown in Luria-Bertani liquid media with 50 μg/mL kanamycin and 20 μg/mL rifampicin at 28 °C in a shaker. Cultures carrying the viral replicon were mixed with separate cultures carrying pCB301-p33, pCB301-94, and pBIN61S-p14. The final concentrations of the cultures at OD_600_ after mixing were: 0.1 for the viral replicon, 0.15 for p33, 0.015 for p94, and 0.1 for p14. Culture mixtures were infiltrated into the 6th true leaf of *Nicotiana benthamiana* plants. Control plants were infiltrated with only infiltration solution (10 mM MgCl_2_, 10 mM MES, 100 µM acetosyringone). After infiltration, plants were grown for 5 to 7 days. Total RNA was extracted from infiltrated leaves using phenol-chloroform and RNA extraction buffer (50 mM Tris-HCl [pH 7.5], 5 mM EDTA [pH 8.0], 100 mM NaCl, 1% SDS), followed by ethanol precipitation. RNA gel blots were performed to assess viral RNA accumulation and p14 mRNA levels. Quantitation of viral replicon band intensities was performed using ImageJ software.

### 2.8. RNA Structure Drawing

All two-dimensional drawings of RNA structures were produced using the on-line version of RNA2Drawer at https://rna2drawer.app (accessed in February through November 2022) [46].

## 3. Results

### 3.1. A Subset of Umbravirus 3′ Terminal Regions Contain Highly Conserved Elements

**Figure 2:**
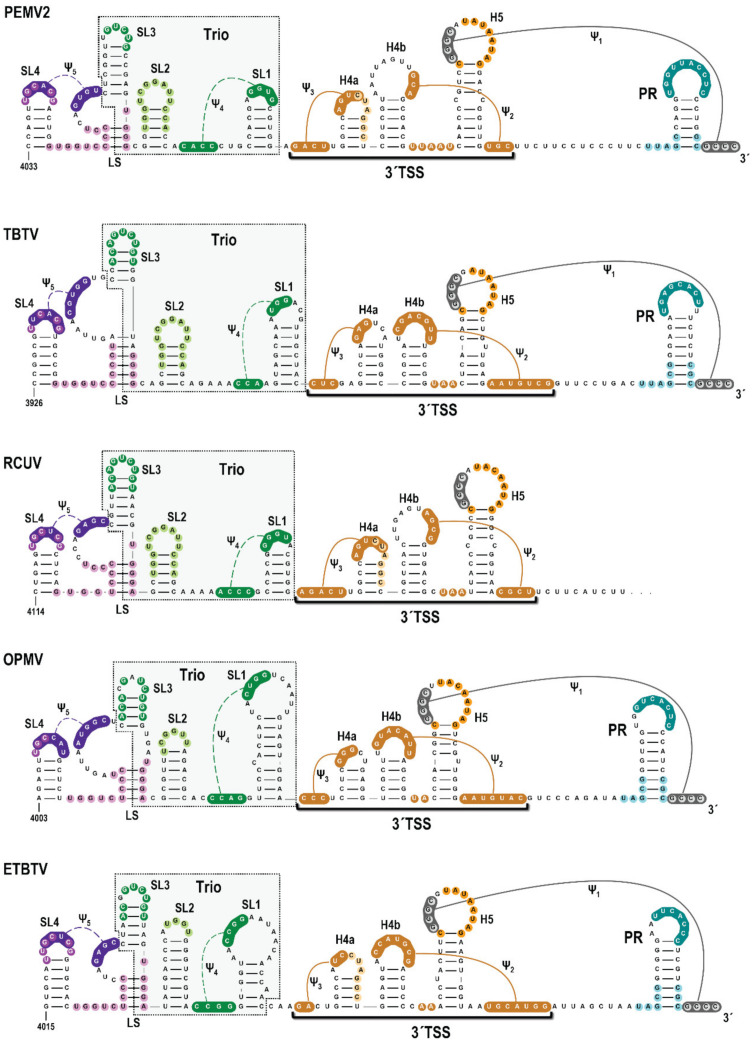
Umbravirus 3′ terminal regions with conserved elements upstream of the 3′TSS. Umbravirus 3′ terminal structures were predicted based on Mfold structure predictions that were modified by folding queries within RNA2Drawer. Conserved sequences are colored alike. Missing 3′end sequence for the reported RCUV sequence is indicated by trailing dots. PR loop sequences that participate in a long-distance interaction to promote ribosome recoding are shaded in teal [11]. Trio elements immediately upstream of 3′TSS structures are boxed.

Examination of the uncharacterized region immediately upstream of the PEMV2 3′TSS in 14 umbraviruses using phylogenetic alignments and Mfold queries [47] revealed that 5 umbraviruses (PEMV2, tobacco bushy top virus [TBTV], red clover umbravirus [RCUV], opium poppy mosaic virus [OPMV], and Ethiopian tobacco bushy top virus [ETBTV]) have sequences capable of forming three putative stem-loops (Figure 2; denoted as SL1, SL2 and SL3 and collectively labeled as “Trio”). SL1, located 0 to 3 nt upstream of the 3′TSS, contains apical loop sequence capable of forming a 3 to 4 bp H-type pseudoknot (Ψ_4_) with nearby upstream sequences. SL2, which is located 0 to 4 A-rich residues upstream of SL1, and SL3, located 5 to 10 G-rich residues upstream of SL2, have high sequence conservation in their apical loops that can extend into the stems. An additional stem-loop (SL4) is located 4 to 6 G/U-rich residues upstream of the SL3 supporting stem. The SL4 apical loop may form a pseudoknot (Ψ_5_) with the bulge loop created by the SL3 supporting stem. Five of the remaining 9 umbraviruses also have SL1/Ψ_4_ in the identical location upstream of the 3′TSS, but are missing SL2, SL3 and SL4 (Figure S1). While SL4 and Ψ_5_ are closely associated with Trio, only Trio elements were examined experimentally for this report.

### 3.2. High-Throughput SHAPE Structure Probing of the 3′UTR of PEMV2 gRNA

**Figure 3:**
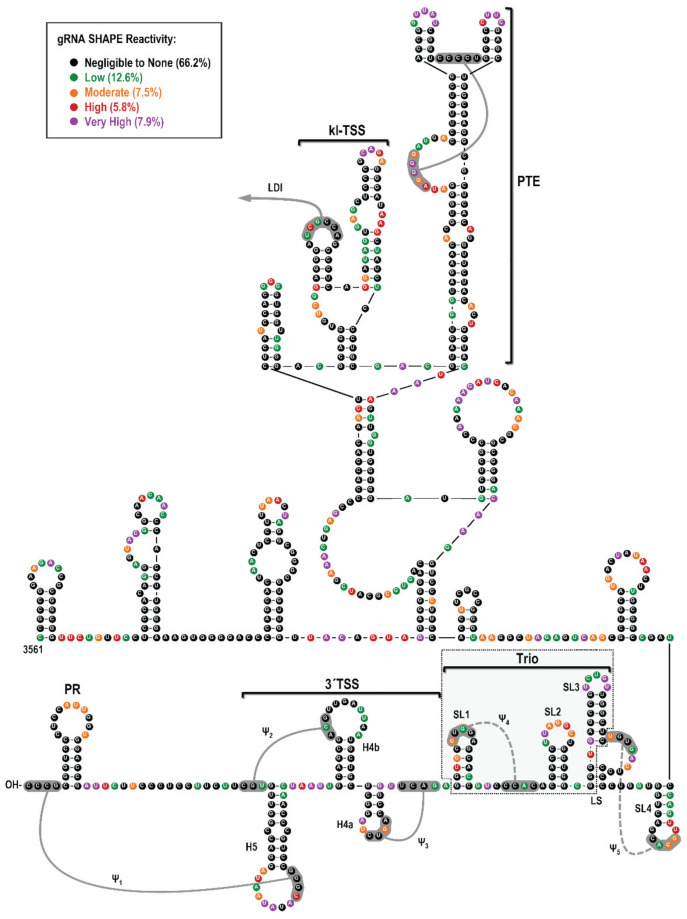
High-throughput SHAPE probing of full-length PEMV2 gRNA. RNA structures previously identified along with Trio are indicated [17,18]. Previously identified pseudoknots have solid lines, while putative pseudoknots have dashed lines. Colored circles around each base indicate average SHAPE reactivity from three independent experiments. Negligible to none (black), low (green), moderate (orange), high (red), and very high (purple). Percentages of residues in each SHAPE reactivity category are indicated. Note that SL1 structure is not supported by SHAPE data.

To begin examining the validity of these conserved structures, SHAPE structure probing [36] was performed on full-length PEMV2 gRNA transcripts synthesized in vitro. Data obtained following primer-extension using fluorescently labeled probes [48,49] were analyzed using QuSHAPE software to quantify cDNA peak sizes obtained by capillary gel electrophoresis [37] (Figure 3). The SHAPE reactivity profile corresponded well with previous SHAPE data attained using radiolabeled primers and traditional gel electrophoresis [17], and supported previously characterized structures, such as the PTE, kl-TSS, 3′TSS and PR [11,18]. SHAPE data were also consistent with SL2, SL3, SL4 and Ψ_4_. However, SHAPE data did not support the presence of SL1 as two residues in the SL1 stem were highly reactive. In addition, the majority of residues proposed to form Ψ_5_ were reactive. Altogether, these results are consistent with SL2, SL3, SL4 and Ψ_4_ existing in the in vitro synthesized PEMV2 gRNA transcripts.

### 3.3. Trio Hairpins Are Critical for PEMV2 Viral Fitness

**Figure 4:**
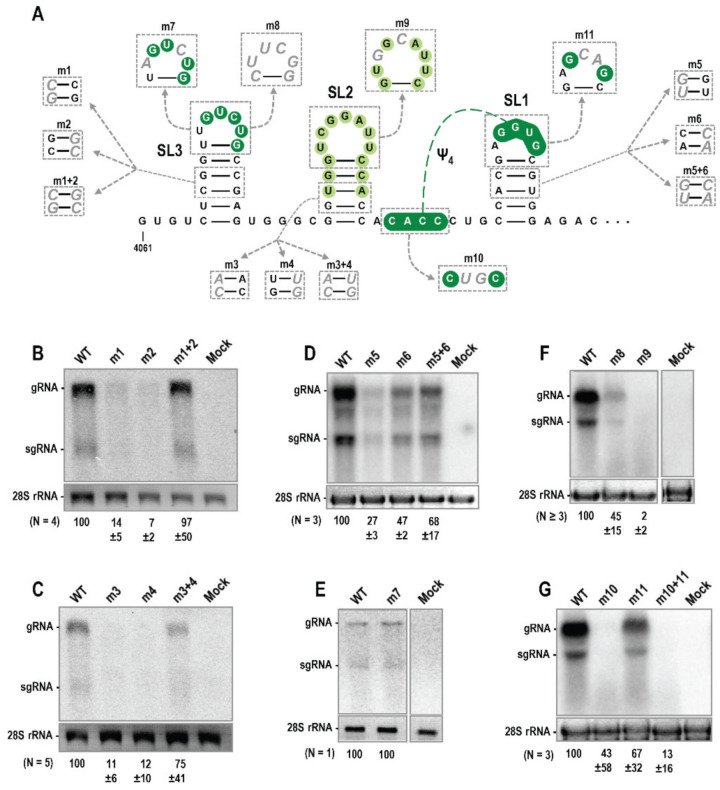
Trio elements are important for PEMV2 accumulation in protoplasts. (**A**) Location of mutations generated within Trio. (**B**–**G**) RNA gel blot analysis of wild-type (WT) PEMV2 accumulation versus with Trio mutations. Mock samples were transfected with transfection solution alone. Ethidium-stained 28S rRNA was used as loading control. Average densitometry measurements of viral gRNA with standard deviations from at least triplicate experiments are shown below the blots with the exception of the experiment illustrated in panel (**E**).

To determine if Trio hairpins are important for PEMV2 accumulation in vivo, two-nucleotide point mutations were generated on both sides of SL1, SL2 or SL3 stems (Figure 4A). Combining point mutations on both sides of the stems would be compensatory and reform the stem. Full-length wild-type (WT) and mutant PEMV2 gRNAs were transfected into protoplasts of *Arabidopsis thaliana* callus cultures and total RNA was extracted 24 h later for RNA gel blot analyses to assess viral RNA accumulation. Mutations on either side of the SL3 stem (m1 and m2) were highly detrimental and reduced gRNA levels to 14% and 7% of WT, respectively (Figure 4B). Combining the two mutations (m1 + 2) restored gRNA accumulation to 97% of WT levels. Mutations disrupting the stem of SL2 (m3 and m4) also had a significant impact, reducing gRNA accumulation to 11% and 12% of WT, respectively (Figure 4C). Combining these two mutations to regenerate the SL2 stem (m3 + 4) restored gRNA accumulation to 75% of WT. Mutations disrupting the stem of SL1 (m5 and m6) were less detrimental, reducing gRNA accumulation to 27% (m5) and 47% (m6) of WT (Figure 4D). Combining m5 + 6 mutations restored gRNA accumulation to 68% of WT. Overall, these results: (1) support the SHAPE and phylogenetic data for SL2 and SL3; (2) provide evidence that SL1 also forms in vivo; and (3) strongly suggest that the Trio stem-loops are important for PEMV2 accumulation during the infection cycle.

Since hairpin apical loop sequences are frequently involved in RNA:RNA or RNA:protein interactions, mutations were also generated in Trio apical loops. Altering two residues in the SL3 loop (m7) did not affect gRNA accumulation (Figure 4E). Considering the high conservation of SL3 loop sequence among umbraviruses (Figure 2), a more extensive alteration that converted the sequence to a highly sTable 5′cUUCGg tetraloop (m8) was also tested. This alteration had a greater impact, reducing gRNA levels to 45% of WT (Figure 4F). A 2-nt alteration in the SL2 apical loop (m9) reduced gRNA levels to 2% of WT, suggesting a critical function for the SL2 loop (Figure 4F). A 2-nt alteration in the loop of SL1 (m11), which should also eliminate the formation of phylogenetically conserved Ψ_4_, had only a modest effect on virus accumulation, reducing levels to 67% of WT (Figure 4G). Mutation of the upstream putative partner sequence (m10) produced unusually inconsistent results, with gRNA levels remaining close to WT (110%) in one replicate and reduced to 9% and 10% of WT in the other two replicates. The combined mutation (m10 + 11) also produced inconsistent results, with gRNA levels reaching 32% of WT and 3% and 5% of WT in the other two replicates. The lack of compensation observed with the combined m10 + 11 mutation suggests either: (1) conservation of this pseudoknot in 10 umbraviruses is fortuitous; (2) the pseudoknot is important to prevent the SL1 apical loop from pairing elsewhere, which is mitigated when the apical loop is mutated. Altogether, these results support an important function for Trio stem-loops that for SL2 involves its highly conserved apical loop.

### 3.4. Alterations in Trio Hairpins Do Not Affect PEMV2 Translation

**Figure 5:**
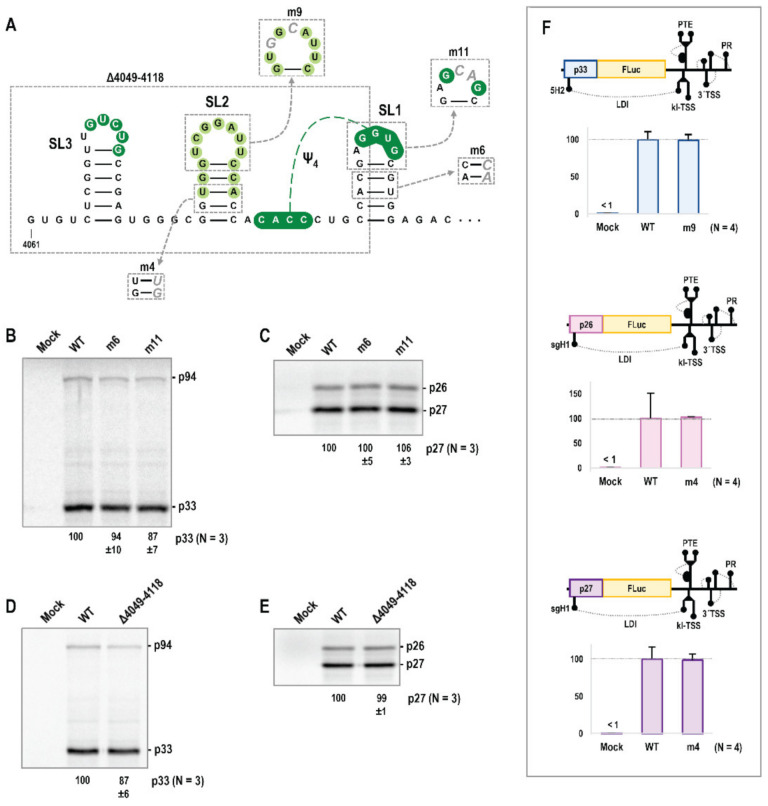
Trio elements are dispensable for gRNA and sgRNA translation. (**A**) Location of Trio mutations. (**B**–**E**) In vitro translation of full-length gRNA or sgRNA. Averages with standard deviation from three independent experiments are shown. (**F**) Translation of luciferase reporter constructs containing the full-length 3′UTR and either the gRNA 5′89 nt (**top**), sgRNA 5′156 nt for p26 (**middle**), or sgRNA 5′157 nt for p27 (**bottom**). Average luminescence readings with standard deviation relative to WT from four independent experiments.

PEMV2 gRNA and sgRNA translation in wheat germ extract (WGE) was previously shown to be negatively affected by deletion of the PTE or kl-TSS 3′CITEs, or by point mutations that eliminated the long-distance interaction between the kl-TSS and the 5′end of the genome [18]. Additionally, PEMV2 sgRNA translation was negatively affected by deletion of the 3′TSS. To determine if SL1 enhances virus accumulation through a role in translation, WGE was programmed with in vitro transcribed full-length PEMV2 gRNA or sgRNA containing point mutations m6 (in the SL1 stem) or m11 (in the SL1 loop). As shown in Figure 5B,C, neither alteration had a significant effect on PEMV2 gRNA or sgRNA translation in WGE. To further examine the Trio stem-loops, a deletion eliminating SL2, SL3 and half of SL1 (Δ4049–4118) was generated and tested for effects on PEMV2 gRNA and sgRNA translation in WGE (Figure 5D,E). Again, no significant effect on translation was found, indicating that these stem-loops do not impact PEMV2 translation in vitro.

Since some 3′CITEs, including the TCV TSS, enhance viral translation in reporter constructs assayed in protoplasts but not in full-length gRNA assayed in WGE [50,51], additional mutations were tested in luciferase reporter constructs containing the full-length PEMV2 3′UTR and sufficient 5′ terminal sequence from the gRNA (5′89 nt) or sgRNA (5′156 nt for p26 translation or 5′157 nt for p27) for efficient translation in vivo [22,41]. m9 (in the SL2 loop) was added to the gRNA luciferase reporter, and m4 (in the SL2 stem) was added to the sgRNA constructs. In vitro transcribed reporter transcripts were transfected into protoplasts prepared from *Arabidopsis thaliana* seed callus cultures and luciferase activity was assayed 18 h later. The SL2 mutations caused no significant effects on luciferase levels in any of the constructs (Figure 5F), suggesting that this critical stem-loop is important for a viral property other than translation.

### 3.5. Trio Stem-Loops Are Important for PEMV2 Replication

**Figure 6:**
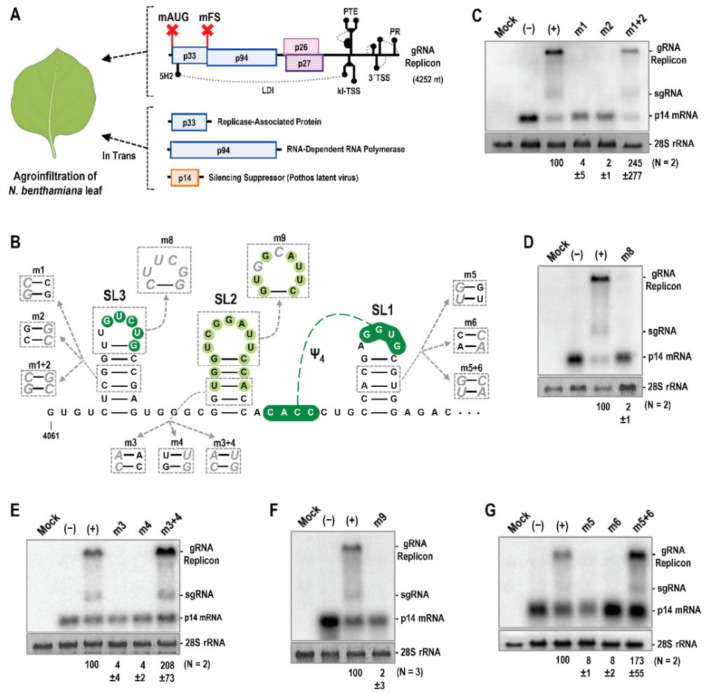
Trio hairpins affect replication of a PEMV2 replicon. (**A**) Replication assay using a full-length PEMV2 gRNA replicon with mutations in p33 initiation codon (mAUG) and the p94 frameshift sequence (mFS) (denoted by red X’s). *N. benthamiana* leaves were agroinfiltrated with the parental replicon and mutant replicon constructs, constructs expressing p33 and p94, and a construct expressing PoLV p14 silencing suppressor. RNA was extracted 5 to 7 days later and probed with radiolabeled oligonucleotides complementary to PEMV2 gRNA and sgRNA 3′ regions and p14 mRNA. (**B**) Trio mutations tested. (**C**–**G**) Data from at least two independent experiments for each set of mutations tested with mean gRNA replicon levels and standard deviations shown.

The proximity of Trio stem-loops to the 3′end of the viral genome suggested possible involvement in (−)-strand synthesis [1]. To test if Trio elements are important for viral replication, a trans-replication system for PEMV2 was developed consisting of a translationally incompetent PEMV2 gRNA replicon and the two PEMV2 replicase proteins synthesized in trans, thus uncoupling viral RNA accumulation from PEMV2 gRNA translation (Figure 6A). PEMV2 gRNA replicon cDNA was generated by mutating the p33 start codon (AUG to ACG) and p94 frameshift slippery sequence (GGAUUUU to GGAUUCU) to eliminate frameshifting [11]. The replicon was introduced into *Agrobacterium tumefaciens* T-DNA with transcription controlled by the Cauliflower mosaic virus 35S promoter and infiltrated into *Nicotiana benthamiana* leaves [45] along with p33 and p94 ORFs supplied on separate T-DNAs, and pothos latent aureusvirus (PoLV) p14 RNA silencing suppressor to reduce degradation of viral RNA [44]. Total RNA was extracted from infiltrated leaves 5 to 7 days later and viral RNA levels were assessed by RNA gel blots (Figure 6C–G). Mutations that disrupted the SL3 stem (m1, m2) and loop (m8) reduced replicon accumulation to 4% of WT or less, whereas the compensatory m1 + 2 mutation enhanced accumulation to at least WT levels (Figure 6C,D). Likewise, the SL2 stem (m3, m4) and loop (m9) mutations reduced replicon levels to 4% of WT or less, with the compensatory m3 + 4 mutation increasing levels to greater than WT (Figure 6E,F). SL1 stem mutations m5 and m6 reduced replicon levels to 8% of WT, with the compensatory m5 + 6 mutation increasing levels to greater than WT. These results provide additional support for the presence and importance of SL1, and strongly suggest that all Trio stem-loops are critical for replication of PEMV2.

## 4. Discussion

Information on umbravirus replication has relied on identifying structures similar to known replication elements present in other tombusvirids. In particular, the high degree of structural similarity between 3′UTR terminal regions of PEMV2 and TCV suggested that overlapping mechanisms of replication would be employed by the two viruses [3,17]. Structural similarities include 3′TSS in nearly identical locations, along with PR and Ψ_1_. These elements are also found near the 3′end of TCV satellite satC, which shares its 3′terminal region with TCV, and their importance for replication of satC has been well established [3]. TSS hairpin H5 and Ψ_1_ are also found in the 3′UTR of TBSV, where they were first characterized (and where H5 is referred to as “SL3”) [9], and appear to be present in most tombusvirids [2].

Our discovery of Trio stem-loops and phylogenetically conserved Ψ_4_ in PEMV2, TBTV, RCUV, OPMV and ETBTV significantly extends our knowledge of umbravirus-specific replication elements. SHAPE data supported the presence of all Trio elements within in vitro synthesized transcripts with the exception of SL1, suggesting that this hairpin may require a conformational rearrangement for formation. We recently found a conserved umbravirus hairpin (the CITE-associated structure; CAS) just upstream of all 3′CITEs located in the middle portion of the 3′UTR (i.e., BTE for OPMV and kl-TSS/PTE for PEMV2) that suppresses a conformational change in OPMV affecting SL1 and formation of the 3′TSS (S. Bera and A.E. Simon, unpublished). Alterations in PEMV2 CAS did not elicit a similar conformational change, suggesting that despite conservation of hairpins from SL4 to the 3′end in PEMV2 and OPMV, interactions of this region with upstream elements differs markedly.

Trio hairpins were critical for both PEMV2 accumulation in Arabidopsis protoplasts (Figure 4) and for replication of a PEMV2 replicon in an agroinfiltration-based trans-replication system in *N. benthamiana* (Figure 6). In contrast, Trio stem-loops were dispensable for translation of full-length gRNA and sgRNA in vitro and reporter constructs in vivo (Figure 5). Although we were unable to confirm the presence of Ψ_4_ in PEMV2 using compensatory mutations (Figure 4G), its association with all 10 SL1 hairpins suggests a conserved function not discernible from these assays. The unusual variation in results for the m10 and m10 + 11 mutations (Figure 4G) may be due to slight differences in the preparation and handling of in vitro transcribed RNA samples between replicate experiments that may have affected the folding of pseudoknotted elements such as SL1 and Ψ_4_ that have potential alternative conformations. The mechanism(s) by which Trio elements support PEMV2 replication (e.g., binding to the viral RdRp or host factors involved in viral replication) also remain to be identified.

A strong correlation was found between relatedness of RdRp nt sequences among umbraviruses and the presence of all Trio elements (Figure 1A), as the 5 umbraviruses containing complete Trio elements (PEMV2, TBTV, RCUV, OPMV, ETBTV) formed a clade with a high confidence probability of 0.965. In the phylogenetic tree based upon 3′UTR sequences (Figure S3), these five umbraviruses also formed a clade with an even higher confidence probability of 1, suggesting that 3′UTR sequences are more closely related among these five umbraviruses compared with RdRp sequences. Interestingly, some umbraviruses lacking some (or all) Trio elements including PSCYV, Changjiang tombus-like virus and GRV were found in the same clade as the five umbraviruses containing all Trio elements. This suggests that the 3′ terminal region of umbraviruses may have undergone virus or host-specific adaptations, as has also been proposed for the more internally located 3′CITE structures of tombusvirids [4], involving loss and possible replacement of Trio elements. Umbraviruses that contain only SL1 (PatMMoV, CMoMV, PasUV1, CMoV, and PicUV1) have varying upstream hairpins, with the exception of closely related CMoV and PasUV1 (Figure S1), and it is possible that these upstream elements provide alternative functional replication-required structures. PSCYV and Changjiang tombus-like virus lack all Trio components including SL1 and deserve examination to determine if this region in these viruses is also important for replication (Figure S2). Furthermore, while the 3′TSS, PR and 5′GCCC-OH motif are generally well conserved in umbraviruses, GRV and IXYMoV have unrelated 3′end sequences and structures (not shown) based on the currently available sequences for these viruses. Despite replication mechanisms appearing to be better conserved relative to mechanisms of translation, divergent mechanisms of replication may also exist among umbraviruses and remain to be explored.

## Data Availability

Not applicable.

## Supplementary Materials

The following supporting information can be downloaded at: https://www.mdpi.com/article/10.3390/v14122615/s1, Figure S1: Umbravirus 3′ terminal structures with incomplete Trio elements; Figure S2: Umbravirus 3′ terminal structures lacking any Trio elements; Figure S3: Maximum likelihood phylogenetic tree for genus Umbravirus based on 3′UTR sequences.
