# Supplementary material for: Novel 3′ Proximal Replication Elements in Umbravirus Genomes
Source: Viruses. 2022 Nov 23;14(12):2615. doi: 10.3390/v14122615 (PMC9780821; doi:10.3390/v14122615)
Supplement: Supplementary file 1 [file viruses-14-02615-s001.zip › viruses-2027345-supplementary.pdf]

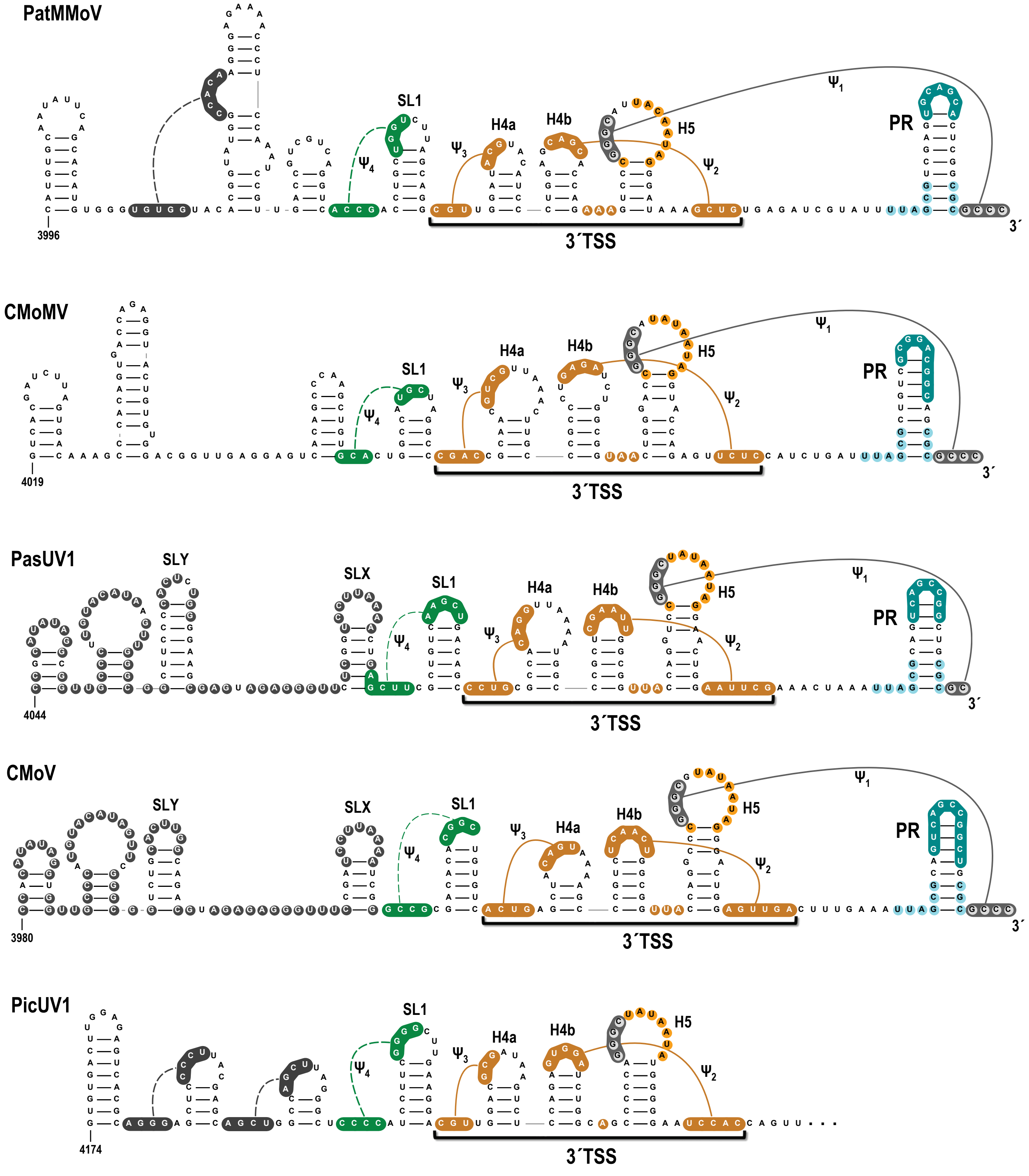

Figure S1. Umbravirus 3´ terminal structures with incomplete Trio elements. All umbraviruses shown have SL1 and Ψ_4 immediately upstream of a complete 3´TSS structure. Color scheme for conserved sequences/pseudoknots is the same as in Fig. 2. Possible pseudoknots upstream of Ψ_4 are colored black. Only CMoV and PasUV1 have similar sequences/structures just upstream of Ψ_4 (conserved residues are circled in black). The current PasUV1 sequence (OL472237.1) terminates unexpectedly with 5´GC and not the full 5´GCCC motif and Ψ_1 found in nearly all umbraviruses. The very 3´ terminus of PicUV1 is not shown (indicated by trailing dots) due to its unusual length and divergent nature (OL472231.1).

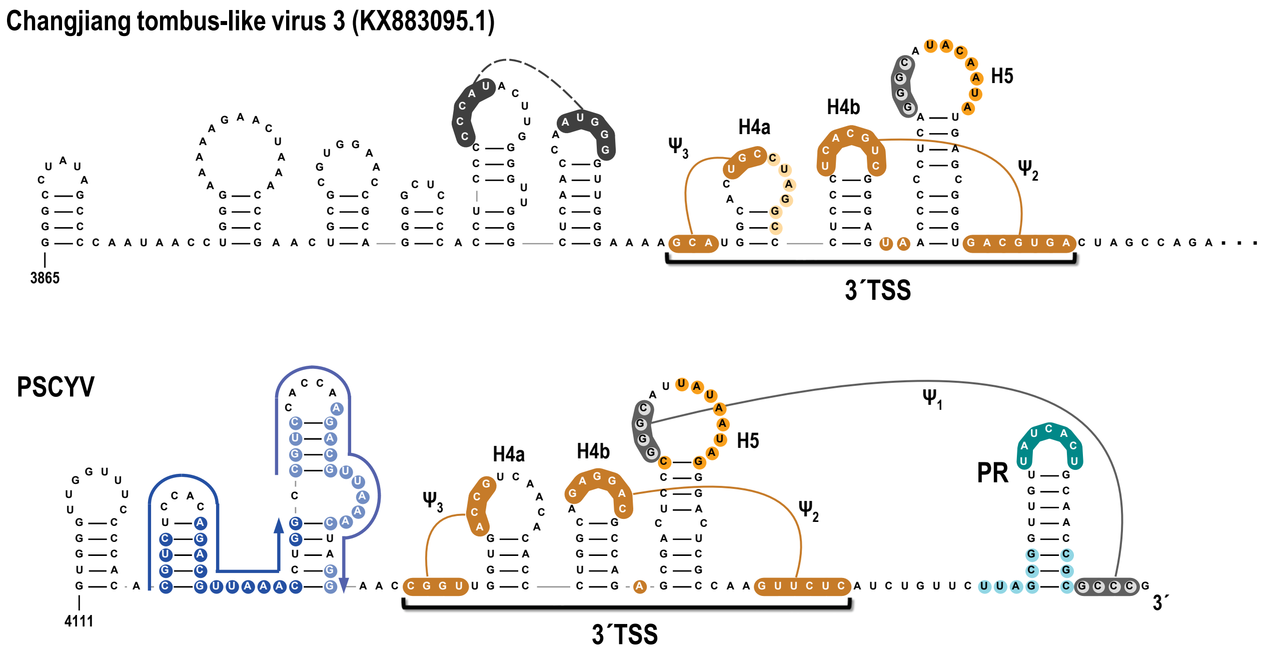

Figure S2. Umbravirus 3´ terminal structures lacking any Trio elements. The color scheme for conserved sequences/pseudoknots is the same as in Fig. 2. Possible pseudoknots upstream of the 3´TSS are colored black. Duplicated sequence just upstream of the 3´TSS in PSCYV is highlighted in blue and light blue. 3´ terminal sequence absent in Changjiang tombus-like virus is indicated by trailing dots. The current PSCYV sequence (OP053684.1) also terminates unexpectedly with a guanylate residue immediately downstream of the 5´GCCC motif and Ψ_1.

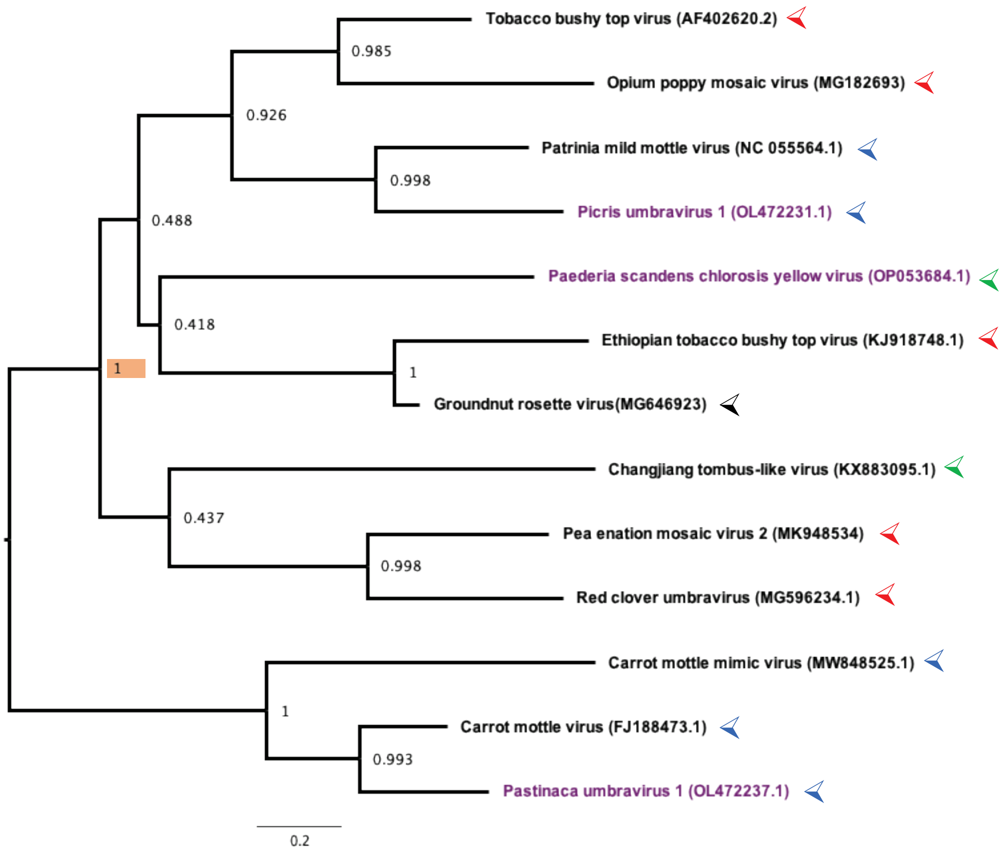

Figure S3. Maximum likelihood phylogenetic tree for Umbravirus genus based on 3´UTR sequences. IXYMoV was omitted from this tree given the unusually short length of its 3´UTR (420 nt; ~700 nt is typical for umbraviruses) in the currently available sequence (KT946712.1). Confidence probabilities for each node are indicated (out of 1,000 bootstraps in building the tree). Scale bar indicates nucleotide substitutions per site. The three recently discovered umbraviruses PSCYV, PicUV1 and PasUV1 are colored purple. Colored carets next to virus names are as described in Fig. 1A. Confidence probability for the nearest ancestral node for the 5 umbraviruses that possess complete Trio elements is highlighted in salmon color.
